# Comparison of the genexpert enterovirus assay (GXEA) with real-time one step RT-PCR for the detection of enteroviral RNA in the cerebrospinal fluid of patients with meningitis

**DOI:** 10.1186/s12985-015-0258-8

**Published:** 2015-02-13

**Authors:** JiYoung Hong, Ahyoun Kim, Seoyeon Hwang, Doo-Sung Cheon, Jong-Hyen Kim, June-Woo Lee, Jae-Hak Park, Byunghak Kang

**Affiliations:** Division of Vaccine Research, Center for Infectious Diseases, National Institute of Health, Korea Center for Disease Control and Prevention, Cheongju, Republic of Korea; Department of Pediatrics, Catholic University College of Medicine, Suwon, Republic of Korea; Department of Laboratory Animal Medicine, College of Veterinary Medicine, Seoul National University, Seoul, Republic of Korea

**Keywords:** GeneXpert Enterovirus Assay, Real-time one step reverse transcription polymerase chain reaction, Enteroviruses, Cerebrospinal fluid

## Abstract

**Background:**

Enteroviruses (EVs) are the leading cause of aseptic meningitis worldwide. Detection of enteroviral RNA in clinical specimens has been demonstrated to improve the management of patient care, especially that of neonates and young children.

**Findings:**

To establish a sensitive and reliable assay for routine laboratory diagnosis, we compared the sensitivity and specificity of the GeneXpert Enterovirus Assay (GXEA) with that of the reverse transcription polymerase chain reaction (RT-PCR) based assay referred to as real-time one step RT-PCR (RTo-PCR). The sensitivity/specificity produced by GXEA and RTo-PCR were 100%/100% and 65%/100%, respectively.

**Conclusions:**

Both methods evaluated in this article can be used for detection of enterovirus in clinical specimens and these nucleic acid amplification methods are useful assays for the diagnosis of enteroviral infection.

## Findings

Enteroviruses (EVs) are the most common cause of aseptic meningitis in children and adults and may cause up to 90% of aseptic meningitis cases [1]. The rapid and accurate diagnosis of human enteroviral infections can reduce the use of antibiotics, duration of hospitalization, and financial costs [2-6]. Methods involving the amplification of nucleic acids have replaced traditional culture-based methods as the gold standard for the detection of EVs in cerebrospinal fluid (CSF) because of their increased sensitivity and speed. Routine diagnostic methods using real-time reverse transcription polymerase chain reaction (RT-PCR) have been developed over the past 15 years in an attempt to improve experts’ ability to detect EVs in CSF [7]. The latest development in the diagnosis of enteroviral meningitis is the GeneXpert Enterovirus Assay (GXEA; Cepheid, Sunnyvale, CA), a fully automated real-time multiplex RT-PCR assay. GXEA is the only assay approved by the U.S. Food and Drug Administration for the qualitative detection of enteroviral RNA in CSF. The GeneXpert system is a closed, unit-dose, molecular, microfluidics instrument that performs extraction, processing, and real-time RT-PCR, as well as the detection of nucleic acid targets. The system uses single-use cartridges that contain all of the reagents required for sample processing and PCR [8-10].

We compared the speed and reliability of GXEA with that of real-time one step RT-PCR (RTo-PCR), a method described in a previous publication by Verstrepen *et al*., that is routinely used for the detection of enteroviral RNA in CSF [11].

To determine the detection range of RTo-PCR using a TaqMan-formatted probe, we obtained five reference strains belonging to different serotypes (enterovirus 71, coxsackievirus B2, echovirus 30, coxsackievirus A24, and poliovirus 1) from the American Type Culture Collection (ATCC) and used these strains to determine the detection limit and positive control of RTo-PCR.

To assess the applicability of GXEA to detect EVs in clinical specimens with a low viral load, from June to September of 2008, we collected 109 CSF specimens from patients with aseptic meningitis. We had approval of an Institutional Review Board (IRB) through the Korean National Institute of Health. We analyzed these specimens by GXEA according to the manufacturer’s instructions using 140 μL of each sample. For the RTo-PCR assay, viral RNA was extracted using silica-coated magnetic beads based on Boom’s method in combination with an automatic liquid handling machine (TECAN, Männedorf, Switzerland) [12]. The TaqMan format RTo-PCR for the detection of EVs was performed according to the previously optimized reaction conditions [11]. Both assays targeted the 5′ noncoding region of the EV genome, which is commonly used in routine diagnostic assays because of its highly conserved genetic identity [11,13].

GXEA and RTo-PCR detected the presence of enterovirus in test samples from five reference strains, of different serotypes, with the limits of detection ranging from 2 to 0.05 TCID_50_/mL for GXEA and from 1 to 0.01 TCID_50_/mL for RTo-PCR (data not shown). Next, we compared the detection efficiency of the recently developed GXEA molecular diagnostic system, which is based on the concept of a “lab on a chip,” with that of RTo-PCR, using the CSF samples collected in 2008 from patients with meningitis. Of the 109 clinical samples assayed by GXEA and RTo-PCR, 66 and 43 were determined to be positive, respectively (Table 1). All of the samples found to be positive by RTo-PCR were also detected by GXEA, with no false positives. In the detection of enteroviral RNA from CSF, the sensitivities/specificities of RTo-PCR and GXEA were 65%/100% and 100%/100%, respectively. The GXEA results were in agreement with those produced by RTo-PCR in 78.9% of cases.

**Table 1:** **Results of RTo-PCR assay and GXEA for 109 clinical samples from patients with aseptic meningitis**

	**No. of positive results/total no. of samples**	**No. of negative results/total no. of samples**
GXEA^*a*^	66/109	43/109
RTo-PCR assay^*b*^	43/109	66/109

^*a*^GeneXpert Enterovirus Assay.

^*b*^Real-time one step RT-PCR.

**Figure 1:**
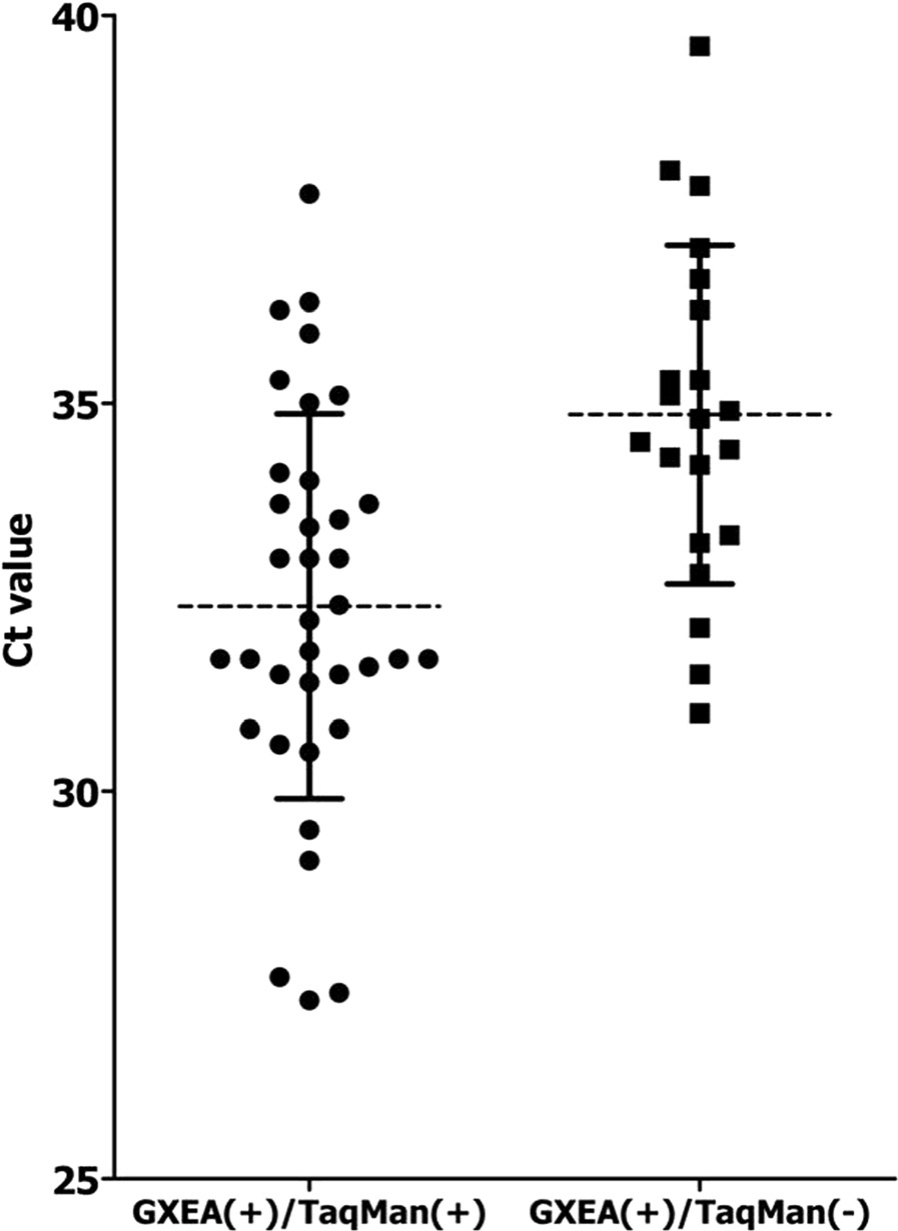
**Comparison of the distributed threshold cycle (Ct) values.** The Ct values of samples identified as positive by both GeneXpert Enterovirus Assay (GXEA) and real-time one-step RT-PCR (RTo-PCR) (GXEA(+)/TaqMan(+):●) and the Ct values of GXEA positive but RTo-PCR assay negative (GXEA(+)/TaqMan(+):■) samples were graphed, respectively. The Ct mean value and standard deviation (S.D.) of double-positive samples was 32.38 and 2.48 respectively. The Ct mean value and S.D. of GXEA positive but RTo-PCR negative samples was 34.85 and 2.18 respectively.

To compare the detection efficiencies of the assays used in this study, we used the mean Ct values from the positive samples; specifically, we divided the values from the GXEA-positive samples by those from the RTo-PCR-positive samples, and the values from the GXEA-positive samples by those from the RTo-PCR-negative samples (Figure 1). The mean Ct values from the GXEA- and RTo-PCR-positive samples (GXEA(+)/TaqMan(+)) and those from the GXEA-positive, RTo-PCR-negative samples (GXEA(+)/TaqMan(−)) were 32.38 and 34.85, respectively. According to our results, GXEA is more sensitive than RTo-PCR. This can be explained by the distribution of the Ct values for the positive specimens, which is an indirect measure of the viral load, as shown in Figure 1.

The sensitivity of GXEA was higher than that of RTo-PCR, and GXEA did not produce discordant positive results when conduced on the RTo-PCR-positive specimens. The mean Ct values for the GXEA-only positive samples (GXEA(+)/TaqMan(−)) were about 2.5 fold higher than those for the samples found to be positive by both assays (GXEA(+)/TaqMan(+)); thus, GXEA is at least 10 times more sensitive than routine RTo-PCR. The discrepancy observed in this study amomg the qualitative results for the CSF samples with a low viral load (n = 20) indicates a higher than expected incidence of CSF specimens from patients with viral meningitis with viral titers below the detection limit of routine molecular assays. Evidently, qualitative tests for the detection of EVs can be influenced by the detection limit of the molecular assay being used.

The GXEA system produced a lower Ct value than RTo-PCR. This may be explained by the fact that GXEA utilizes single-use cartridges that contain all of the reagents required for sample processing. Thus, the entire amount of extracted RNA is used in GXEA, while, in RTo-PCR, the amount of RNA used may be 10-fold lower. The sensitivity of an assay depends in part on the total amount of RNA used.

### Viruses, controls, and clinical samples

Five reference strains belonging to distinct genotypes [enterovirus 71 (E71), coxsackievirus B2 (CVB2), echo 30 (E30), coxsackievirus A24 (CVA24), and poliovirus type 1 (P1)] were obtained from the American Type Culture Collection (ATCC). Infectivity of viruses was assayed by microplates in serial 10-fold dilutions (from 10^-4^ to 10^-10^) in quadruplicate (four wells per dilution). TCID_50_ titers were calculated according to the Kärber method [14]. In total, 109 clinical specimens were collected from patients with suspected viral meningitis between June and September 2008.

### Extraction of viral RNA

RNA was extracted from 150 μL samples with the GM Viral Nucleic Acid Extraction Kit (GreenMate Biotech Corp, Korea), according to the manufacturer’s protocol, using automated machines for liquid handling (Tecan, Switzerland). The GM Viral Nucleic Acid Extraction Kit uses a silica-based extraction method [12]. RNA was then recovered in 50 μL of nuclease-free water. It was used immediately or stored at -70°C.

### One-step real-time RT–PCR

Real-time one step RT-PCR (RTo-PCR) was performed using an ABI Prism 7900HT sequence detection system (Applied Biosystems). Viral RNA was amplified in 25 μL reactions using RT–PCR master mix (AgPath-ID one-step RT–PCR Kit; Ambion, CA). Reactions were incubated at 45°C for 15 min, and then at 95°C for 10 min, followed by 45 cycles of 95°C for 15 s and 60°C for 40 s.

Viral nucleic acid amplification is being developed as a diagnostic procedure to detect enteroviral infections [15]. There is an urgent need to devise standardized methods, utilizing commercialized kits, that can supplement or replace the diagnostic tests that are often developed in-house, and that are, therefore, inconsistent in their sensitivity and specificity. The GeneXpert machine and assay kit, which has been approved by the U.S. Food and Drug Administration for the detection of EVs, produces rapid and highly sensitive results from CSF samples [9,10].

These innovative tests, based on state-of-the-art technologies, provide the means by which to achieve more accurate clinical laboratory results and, therefore, better patient care, when attempting to diagnose or rule out sepsis.

## Abbreviations

EVs: Enteroviruses
GXEA: GeneXpert enterovirus assay
RTo-PCR: Real-time one step RT-PCR
CSF: Cerebrospinal fluid
IRB: Institutional review board
E71: Enterovirus 71
CVB2: Coxsackievirus B2
E30: Echo virus 30
CVA24: Coxsackievirus A24
P1: Poliovirus type 1
ATCC: American type culture collection
